# Triphenylamine-Containing Benzoic Acids: Synthesis, Liquid Crystalline and Redox Properties

**DOI:** 10.3390/molecules28072887

**Published:** 2023-03-23

**Authors:** Beatriz Feringán, Alejandro Martínez-Bueno, Teresa Sierra, Raquel Giménez

**Affiliations:** Instituto de Nanociencia y Materiales de Aragón (INMA), Departamento de Química Orgánica, Facultad de Ciencias, CSIC-Universidad de Zaragoza, 50009 Zaragoza, Spain

**Keywords:** triphenylamine, liquid crystal, redox, electroactive, photoactive, hole transport, organic semiconductor, hydrogen bond

## Abstract

The synthesis, characterization and liquid crystalline and electrochemical properties of novel triarylamines, in which the triphenylamine platform is non-symmetrically modified with a 4-(6-oxyhexyloxy)benzoic acid group, are reported. Compounds show columnar liquid crystalline behavior, as confirmed through the use of polarized optical microscopy, differential scanning calorimetry and X-ray diffraction. Electrochemical properties were measured using cyclic voltammperometry, obtaining low oxidation potentials and HOMO values that were optimum for consideration as organic semiconductors in hole transport layers. In addition, the photoredox activity of one of these derivatives in dichloromethane was studied under light irradiation. A photooxidation/assembly process under white light irradiation occurs without the assistance of hydrogen bonding amide functional groups.

## 1. Introduction

Triphenylamine is a photo and electroactive unit with an electron-donor character [1,2,3]. The wide possibilities of organic synthesis allow the tuning of this electronic property through derivatization, leading to a great variety of compounds that incorporate the triphenylamine platform, in the search for novel hole transport and hole injection materials [4]. Efficient hole transport materials are required in organic electronic devices such as OLEDs and OFETs [5], as well as for organic, dye-sensitized and perovskite solar cells [6,7,8,9]. In all these devices, hole transport materials are usually processed as amorphous layers [10].

Nevertheless, a way to control the electronic communication between functional units is their self-assembly into nanostructures [11,12], and this can be assisted by liquid crystalline (LC) behavior [13,14,15,16,17,18]. Thus, the self-assembly of triphenylamine derivatives in columnar mesophases has led to very high hole mobility values [19].

Despite this, LC triarylamines have barely been described, as their non-planar structure does not favor the formation of LC phases [20]. Some examples report LC behavior in molecules in which triphenylamine is the central core of a *C*_3_ star-shaped molecule, either substituted with phenylethynyl groups [21] or substituted with three conjugated two-aromatic-ring promesogenic substituents and nine terminal chains, in order to favor columnar mesomorphism [22,23,24]. With a different design, the symmetric substitution with arylaminomethyl substituents with four or six terminal tails [25] or asymmetric substitution with hydroxymethyl moiety [25] has also led to columnar mesophases. In a different report, when triphenylamine is non-symmetrically substituted with amide and hydroxybenzyl, substituents show lamellar or columnar mesophases depending on the terminal alkoxy substitution, with five, six or nine terminal chains [26]. In addition, adopting a novel concept, we recently reported on stable hexagonal columnar organizations in which triphenylamine units organize at the periphery of the columns, and this was achieved by supramolecular complexes made by hydrogen bonding between a tris(triazolyl)triazine core and three peripheral triphenylamine containing benzoic acids [19].

**Scheme 1:**
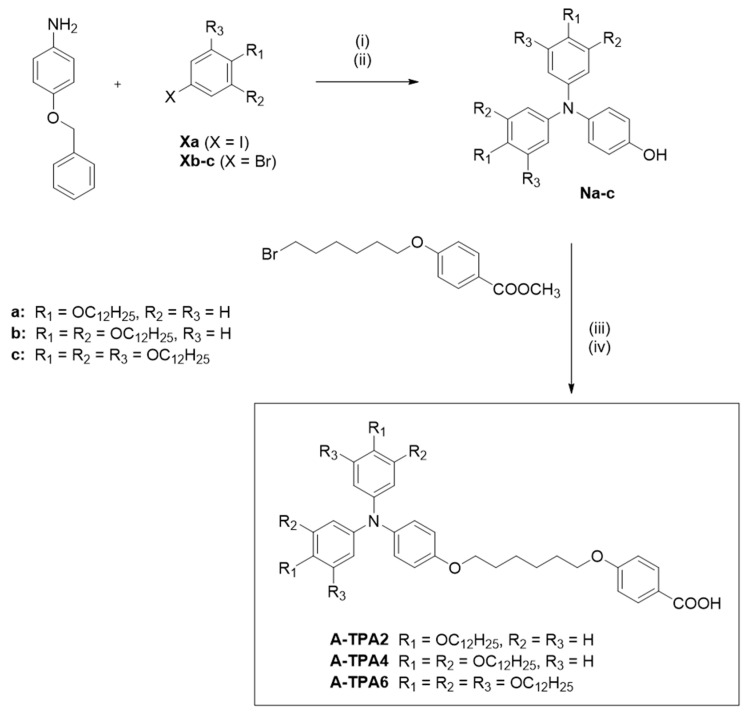
Synthetic route for the preparation of the novel triphenylamine-containing benzoic acids: (**i**) Pd_2_(dba)_3_, P(^t^Bu)_3_, ^t^BuOK, toluene. Yield: 79–83%; (**ii**) H_2_, Pd/C, THF. Yields of Na–**c**: 90–93%; (**iii**) K_2_CO_3_, KI, DMF. Yields of methyl esters Ea–c: 66–85%; (**iv**) (1) KOH, dioxane, H_2_O, (2) HCl_(aq)_. Yields of acids A-TPA_n_: 69–78%.

In this work, we report on novel triphenylamine-containing benzoic acids (Scheme 1, compounds A-TPA2, A-TPA4 and A-TPA6), which can seldom display columnar mesomorphism, and the study of their electrochemical properties. In addition, the photoredox activity of A-TPA2 in dichloromethane is demonstrated through light irradiation experiments in solution.

## 2. Results and Discussion

### 2.1. Synthesis and Characterization

Compounds A-TPA2, A-TPA4 and A-TPA6 were prepared following the synthetic routes depicted in Scheme 1. Polyalkoxylated triphenylamines with a hydroxyl group (Na–c) were prepared by means of a Buchwald–Hartwig coupling between 4-benzyloxyaniline and aryl halides (Xa–c) [19]. Afterwards, the obtained triarylamines Na–c reacted with methyl 4-(6-bromohexyloxy)benzoate in a Williamson-type reaction, then the ester group was hydrolyzed with KOH to potassium carboxylate and acidified to obtain the carboxylic acid. Final compounds were characterized using elemental analysis, mass spectra and ^1^H and ^13^C NMR performed in deuterated dichloromethane in the absence of light. All data were consistent with the proposed structures (see Section 3.2).

### 2.2. Liquid Crystalline Properties

**Figure 1:**
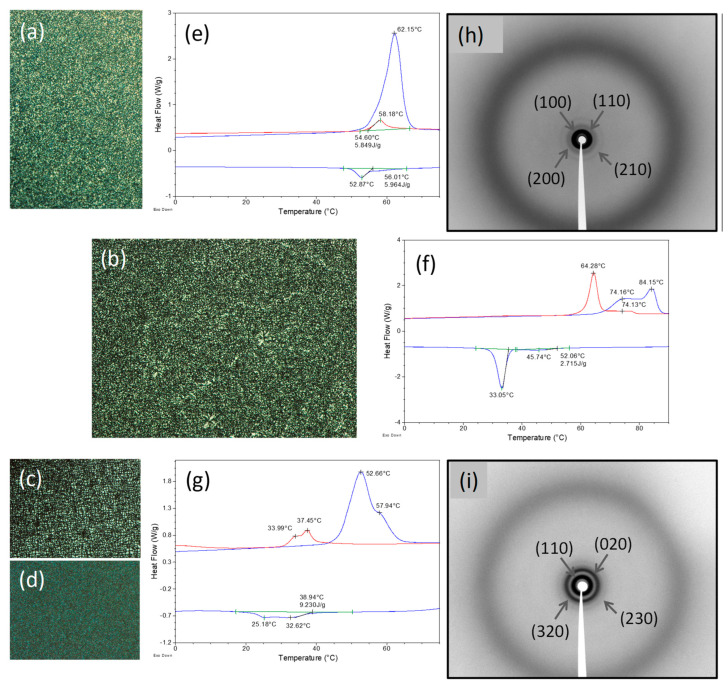
(**a**–**d**) Textures of the liquid crystalline phases observed between crossed polarizers in the cooling cycle for (**a**) A-TPA2 at 52 °C, (**b**) A-TPAC4 at 45 °C, (**c**) A-TPA6 at 28 °C, (**d**) A-TPA6 at 20 °C. (**e**–**g**) DSC thermograms recorded at 10 °C min^−1^. First heating and cooling cycle (blue) and second heating (red) for (**e**) A-TPA2, (**f**) A-TPA4 and (**g**) A-TPA6. (**h**,**i**) XRD diffractograms recorded at 20 °C after cooling from the isotropic liquid for (**h**) A-TPA2 and (**i**) A-TPA6.

Triphenylamine-containing benzoic acids were characterized using POM and DSC (Table 1, Figure 1a–g). All compounds were isolated from the synthesis as crystalline solids that directly melted into the isotropic liquid. Upon cooling, all of them show liquid crystalline properties. From the study of the thermograms, first and second heating cycles were different, and the cooling cycles were reproducible.

A-TPA2 was obtained from the synthesis as a crystalline material that melts at 62 °C and displays a columnar LC phase upon cooling at 53 °C. This LC phase is stable at room temperature. In the second heating cycle, only the transition from the liquid crystal to the isotropic liquid is observed at 58 °C.

In contrast, compounds with a higher number of alkoxy chains, A-TPA4 and A-TPA6, show complex crystalline polymorphism in the heating cycles. A-TPA4 was obtained from the synthesis as a mixture of two crystalline polymorphs that melt into the isotropic liquid at 84 °C. Upon cooling, it shows a LC phase at 45 °C, with a texture compatible with a columnar mesophase, and it rapidly crystallizes at 33 °C. In the second heating cycle, the transition to the isotropic liquid shows peaks at 64 °C and a small broad peak at 74 °C, consistent with the high tendency of this compound to display crystalline polymorphism. A-TPA6 also crystallizes as a mixture of polymorphs and transforms into the isotropic liquid at 58 °C. Upon cooling, it shows two LC phases at 33 °C and 25 °C, which appear at the DSC as a complex overlapped transition in the cooling cycle and the subsequent heating cycle.

The LC phases of A-TPA2 and A-TPA6 could be studied using XRD in samples cooled from the isotropic liquid at room temperature, in accordance with the particular thermal behavior observed at DSC. In both diffractograms, a diffuse halo at wide angles typical of the liquid crystalline state is observed (indicated as diff in Table 2). For A-TPA2, the diffractogram shows a set of four reflections in the low angle region, keeping the relationship of 1:1/√3:1/√4/:1/√7, and this confirms a hexagonal columnar mesophase (Figure 1h, Table 2). For A-TPA6, only the phase at room temperature could be measured. In this case, the diffractogram at low angle does not fit the hexagonal symmetry but, rather, a rectangular symmetry (Figure 1i, Table 2). The high tendency for crystallization of A-TPA4 precluded the study of its LC phase.

The lattice parameters obtained for the LC phases of A-TPA2 and A-TPA6 are large, and they are consistent with the fact that the columnar mesophase is not formed by the stacking of a single molecule, but by aggregates of several molecules. For a comparison with the previously described triphenylamine-containing columnar liquid crystals [19], an estimation can be made, considering similar parameters for density (1 g cm^−3^) and thickness of the columnar stratum (3.4 Å). This yields an average number of seven molecules for A-TPA2 and four molecules for A-TPA6.

**Figure 2:**
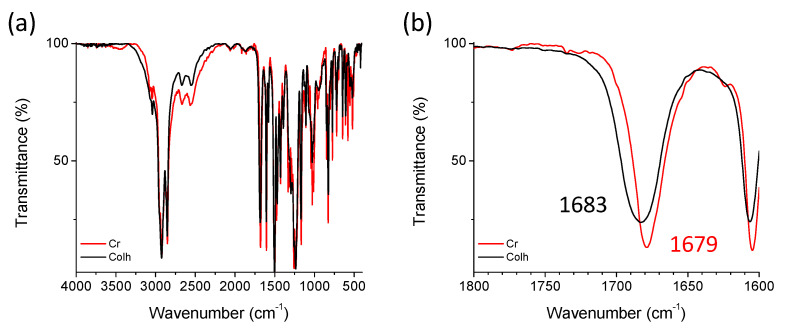
IR spectra for A-TPA2 in the crystal phase (red) and in the hexagonal columnar mesophase (black). (**a**) Full recorded spectra. (**b**) Detail of the C=O stretching band.

*p*-Alkoxybenzoic acids are known to form dimers by hydrogen bonding in the liquid crystalline state [27]. In order to know whether this organization exists in these materials, IR spectra were measured in KBr pellets, in the crystal and columnar LC phases of A-TPA2 (Figure 2). The spectra show an O-H stretching broad band at 3500–2400 cm^−1^ and C=O stretching bands at 1679 or 1683 cm^−1^ for the crystal and LC phases, respectively; typical of carboxylic acids associated with hydrogen bonding. The C=O band found for the crystal phase is assigned to closed dimer entities. In case of the LC phase, the C=O stretching band is broader and the maximum slightly shifts to a higher wavenumber, which points to the heterogeneity of the species, such as some presence of open dimers participating as H-bond acceptors, in addition to the main closed dimer form, in accordance with IR spectra studies of liquid crystalline *p*-alkoxybenzoic acids [28].

Based on this, a self-assembly model involves the formation of aggregates through hydrogen bonding, mainly dimeric, which assemble in columns with the benzoic acids segregated at the center of the disc and the triarylamine and terminal chains organized at the periphery.

### 2.3. Electrochemical Properties

In order to evaluate the redox properties of the novel compounds, the triphenylamine-containing benzoic acids were characterized using cyclic voltammperometry in deaerated dichloromethane solutions in a three-electrode cell, with a carbon disc as working electrode, a Pt wire as counter electrode and the Ag/AgCl electrode as reference. The solutions were 10^−4^ M in the electroactive material; 0.1 M in tetrabutylammonium hexafluorophosphate was used as the supporting electrolyte. Ferrocene/ferrocenium ion redox couple (Fc/Fc⁺) was used as the internal standard.

**Figure 3:**
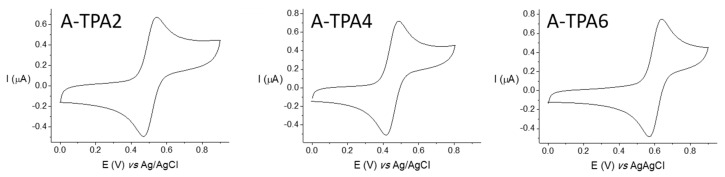
Cyclic voltamperograms corresponding to the first oxidation potential for the synthesized triphenylamine-containing benzoic acids.

In the obtained cyclic voltammperograms, the first oxidation potential appeared as a reversible process (Figure 3), and this corresponds with the oxidation of the triphenylamine units to their radical cation [29]. The half wave potential value is between 0.46 V and 0.60 V vs. Ag/AgCl (Table 3). The alkoxy chain substitution, with electron-donor character, lowers the oxidation potential with respect to the non-substituted triphenylamine unit [30]. On the other hand, the number of alkoxy substituents slightly influences the oxidation process [31]. A-TPA2 has higher oxidation potential than that of A-TPA4, which has one additional alkoxy group at *metha* position in two aromatic rings, and lower than that of the highly substituted A-TPA6 compound. Therefore, the formation of the radical cation is dependent on the substitution. HOMO levels calculated from these data fit well with those reported for organic semiconductors used in hole injection and hole transport layers [2].

### 2.4. Photoactivity in Solution

Some triarylamine derivatives display a photoredox/assembly effect under white light irradiation in chlorinated solvents [32]. This process has been reported for triarylamines substituted with at least one amide group [26,32,33,34,35,36,37]. The triarylamines prepared in this work are substituted with alkoxy side chains but do not have amide groups in their structure. Despite this, we confirm here that A-TPA2 is also photoactive under white light irradiation.

**Figure 4:**
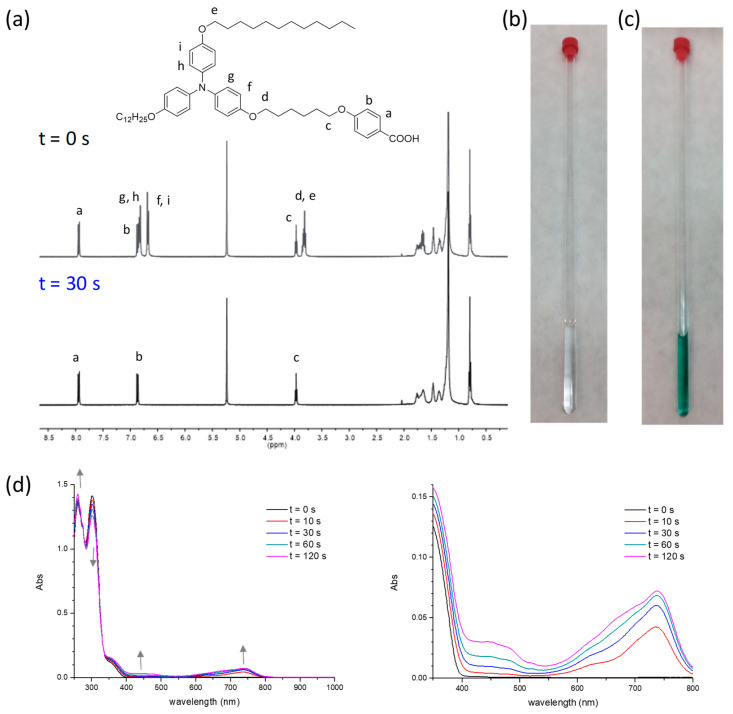
Spectral changes of A-TPA2 under light irradiation. (**a**) ^1^H NMR spectra in CD_2_Cl_2_ and pictures of the NMR tube (**b**) before and (**c**) after irradiation for 30 s. (**d**) UV–Vis–NIR spectra in CH_2_Cl_2_ by increasing the irradiation time (at two different scales).

The light-induced changes were studied using ^1^H-NMR. A solution of A-TPA2 in deuterated dichloromethane (8.9 mM) was measured before and after irradiation, using a 50 W white light dichroic lamp (0.47 mW cm^−2^). The ^1^H-NMR spectrum contains all the signals corresponding to the protons of the pure product when the solution is kept in the dark (Figure 4a, t = 0 s). However, after 30 s of irradiation, the disappearance of the signals corresponding to the protons of the triphenylamine unit (Hf, Hg, Hh and Hi, Figure 4a, t = 30 s) and, also, of the protons of the methyleneoxy substituents was observed. (Hd and He, Figure 4a, t = 30 s). The disappearance of these signals in the ^1^H-NMR spectrum is similar to that described by Giuseppone et al. [26,32] and is explained by the formation of aggregates by the oxidation of some triphenylamine units to their radical cation, giving rise to supramolecular triphenylamine polymers with delocalized radicals, so that the combination of the stacking of the triphenylamine units with the paramagnetic contribution of the radicals causes the disappearance of these signals. The presence of chlorinated solvents is necessary for this process to occur, since the triphenylamine units transfer electrons to the solvent, releasing chloride ions. Furthermore, this oxidation process is accompanied by a color change in the solution, which is transparent before irradiation and becomes green-blue after irradiation (Figure 4b,c). On the contrary, the ^1^H-NMR spectrum in acetone shows no changes after irradiation, confirming that the presence of chlorinated solvents is necessary for the light-induced oxidation.

The color change that accompanies the oxidation process was also studied using UV–Vis–NIR spectroscopy in dichloromethane (5.5 × 10^−5^ M). The absorption spectra obtained after irradiation (Figure 4d) are also in agreement with the formation of the radicals that give rise to the self-assembly process. Before irradiation, the spectrum shows bands in the UV region at 260, 301 and about 350 nm, corresponding to the neutral form of the triphenylamine benzoic acids. Above 400 nm, no band is observed. When irradiated with white light, an absorption band grows at 738 nm, due to the formation of triphenylamine radicals [38]. The intensity of this band increases with irradiation time, as do bands located around 450 nm, due to the stacking of the triphenylamine units induced by the radical formation, confirming that the light-induced self-assembly process occurs [26,32]. The presence of these bands upon irradiation is consistent with the color change of the solution from colorless to green-blue. The UV–Vis–NIR absorption spectra of the non-irradiated solution show that neither additional bands nor color change; thus, light irradiation is necessary for the processes of radical formation and self-assembly of triphenylamine units. In essence, the reported photoactivity for amide-containing triphenylamines also occurs in the absence of amide functional groups in the molecular structure.

## 3. Materials and Methods

### 3.1. Experimental Techniques

All reagents and solvents were purchased from Aldrich or Fisher Scientific and used without further purification. Anhydrous THF was purchased from Scharlab and dried by using a PureSolv solvent purification system. ^1^H-NMR and ^13^C-NMR spectra were acquired using a Bruker AV400 spectrometer. The experiments were performed at room temperature. Chemical shifts are given in ppm relative to the solvent residual peak (CD_2_Cl_2_, δ_H_ = 5.32 ppm, δ_C_ = 54 ppm; CD_3_COCD_3_, δ_H_ = 2.05 ppm, δ_C_ = 29.84 ppm). UV–Vis–NIR spectra were measured in an Agilent Cary 6000i apparatus in double beam mode, using 10 mm path length quartz cuvettes. IR spectra were recorded on a Bruker Vertex 70 FT-IR spectrometer. The samples were prepared using KBr pellets, with a product concentration of 1% (*w/w*). Mass spectra were obtained using a MICROFLEX Bruker (MALDI+) spectrometer with a dithranol matrix. Elemental analyses were performed using a Perkin–Elmer 2400 series II microanalyzer. The mesophases were examined using polarizing optical microscopy, with the polarizing optical microscope Olympus BX51 connected to a Linkam THMS600 hot stage and a Linkam TMS94 controller. Microphotographs of the textures were obtained using an Olympus DP12 digital camera connected to the microscope, using a 20× objective. Transition temperatures and enthalpies were obtained through differential scanning calorimetry with DSC TA instruments Q20 and Q2000, at a rate of 10 °C min^−1^ in a nitrogen atmosphere and in sealed aluminum pans. The instruments were calibrated with indium (T_onset_ = 156.6 °C, ΔH = 28.71 J g^−1^). Powder X-ray experiments were performed in a Pinhole diffractometer (Anton Paar) operating with a point focused Ni-filtered Cu-Kα beam. The samples were held in Lindemann glass capillaries (0.7 and 0.9 mm diameter) and heated with a variable-temperature attachment. The diffraction patterns were collected on photographic films. Cyclic voltammetry experiments were performed using an Autolab PGSTAT204 potentiostat using a three-electrode cell, as described in Section 2.3.

All reagents and solvents were purchased from Aldrich or Fisher Scientific and used without further purification. Anhydrous THF was purchased from Scharlab and dried by using a PureSolv solvent purification system. ^1^H-NMR and ^13^C-NMR spectra were acquired using a Bruker AV400 spectrometer. The experiments were performed at room temperature. Chemical shifts are given in ppm relative to the solvent residual peak (CD_2_Cl_2_, δ_H_ = 5.32 ppm, δ_C_ = 54 ppm; CD_3_COCD_3_, δ_H_ = 2.05 ppm, δ_C_ = 29.84 ppm). UV–Vis–NIR spectra were measured in an Agilent Cary 6000i apparatus in double beam mode, using 10 mm path length quartz cuvettes. IR spectra were recorded on a Bruker Vertex 70 FT-IR spectrometer. The samples were prepared using KBr pellets, with a product concentration of 1% (*w/w*). Mass spectra were obtained using a MICROFLEX Bruker (MALDI+) spectrometer with a dithranol matrix. Elemental analyses were performed using a Perkin–Elmer 2400 series II microanalyzer. The mesophases were examined using polarizing optical microscopy, with the polarizing optical microscope Olympus BX51 connected to a Linkam THMS600 hot stage and a Linkam TMS94 controller. Microphotographs of the textures were obtained using an Olympus DP12 digital camera connected to the microscope, using a 20× objective. Transition temperatures and enthalpies were obtained through differential scanning calorimetry with DSC TA instruments Q20 and Q2000, at a rate of 10 °C min^−1^ in a nitrogen atmosphere and in sealed aluminum pans. The instruments were calibrated with indium (T_onset_ = 156.6 °C, ΔH = 28.71 J g^−1^). Powder X-ray experiments were performed in a Pinhole diffractometer (Anton Paar) operating with a point focused Ni-filtered Cu-Kα beam. The samples were held in Lindemann glass capillaries (0.7 and 0.9 mm diameter) and heated with a variable-temperature attachment. The diffraction patterns were collected on photographic films. Cyclic voltammetry experiments were performed using an Autolab PGSTAT204 potentiostat using a three-electrode cell, as described in Section 2.3.

### 3.2. Synthesis of the Triphenylamine-Containing Benzoic Acids

A round-bottom flask is charged with the corresponding compound Na–c [19] (1.1 mmol), dry K_2_CO_3_ (6.4 mmol, 0.88 g), KI (0.4 mmol, 0.06 g) and 50 mL of dry DMF. Next, methyl 4-(6-bromohexyloxy)benzoate [39] (1.3 mmol, 0.40 g) is added. The reaction mixture is heated to 100 °C for 16 h. It is then poured into 150 mL of distilled H_2_O and extracted with a mixture of hexane/ethylacetate 1/1 (2 × 50 mL). The organic layers are combined and washed with brine, dried over anhydrous MgSO_4_ and filtered. The solution is evaporated to dryness and the residue purified using column chromatography in silica gel to yield the methyl esters Ea–c, as indicated in each particular case.

Ea: Eluent: hexane and then increasing polarity to hexane/ethyl acetate 20/1. Yield: 66%. ^1^H-NMR (400 MHz, CD_3_COCD_3_), δ (ppm): 7.98–7.91 (m, 2H), 7.05–6.98 (m, 2H), 6.94–6.87 (m, 6H), 6.86–6.79 (m, 6H), 4.11 (t, *J* = 6.5 Hz, 2H), 3.97 (t, *J* = 6.4 Hz, 2H), 3.94 (t, *J* = 6.5 Hz, 4H), 3.83 (s, 3H), 1.87–1.70 (m, 8H), 1.61–1.53 (m, 4H), 1.52–1.22 (m, 36H), 0.88 (t, *J* = 6.8 Hz, 6H). ^13^C-NMR (100 MHz, CD_3_COCD_3_), δ (ppm): 166.9, 164.0, 155.6, 155.5, 142.8, 142.7, 132.2, 125.6, 125.5, 123.2, 116.0, 116.0, 115.1, 68.9, 68.8, 68.7, 52.0, 32.7, 30.4, 30.4, 30.4, 30.1, 30.1, 26.8, 26.6, 26.5, 23.3, 14.4. IR (KBr, cm^−1^): 2924 (Csp^3^-H), 2853 (Csp^3^-H), 1721 (C=O), 1508 (arC-C), 1503 (arC-C), 1469 (arC-C), 1236 (C-O).

Eb: Eluent: hexane and then increasing polarity to hexane/ethyl acetate 15/1. Yield: 71%. ^1^H-NMR (400 MHz, CD_3_COCD_3_), δ (ppm): 7.98–7.91 (m, 2H), 7.03–6.98 (m, 2H), 6.98–6.93 (m, 2H), 6.86–6.78 (m, 4H), 6.64 (d, *J* = 2.6 Hz, 2H), 6.47 (dd, *J* = 8.6, 2.6 Hz, 2H), 4.11 (t, *J* = 6.5 Hz, 2H), 3.98 (t, *J* = 6.4 Hz, 2H), 3.95 (t, *J* = 6.4 Hz, 4H), 3.85 (t, *J* = 6.3 Hz, 4H), 3.83 (s, 3H), 1.91–1.65 (m, 12H), 1.63–1.24 (m, 76H), 0.89 (t, *J* = 6.9 Hz, 12H). ^13^C-NMR (100 MHz, CD_3_COCD_3_), δ (ppm): 167.0, 164.1, 156.0, 151.1, 146.1, 143.6, 142.5, 132.3, 126.3, 123.5, 116.8, 116.6, 116.1, 115.2, 111.8, 70.6, 69.9, 69.0, 68.9, 52.0, 32.7, 32.7, 30.5, 30.5, 30.5, 30.5, 30.5, 30.3, 30.2, 30.2, 30.2, 30.1, 29.9, 27.0, 26.9, 26.7, 26.6, 23.4, 14.4. IR (KBr, cm^−1^): 2919 (Csp^3^-H), 2850 (Csp^3^-H), 1726 (C=O), 1509 (arC-C), 1504 (arC-C), 1468 (arC-C), 1239 (C-O).

Ec: Eluent: hexane and then increasing polarity to hexane/ethyl acetate 15/1. Yield: 85%. ^1^H-NMR (400 MHz, CD_3_COCD_3_), δ (ppm): 7.97–7.92 (m, 2H), 7.06–6.97 (m, 4H), 6.90–6.83 (m, 2H), 6.27 (s, 4H), 4.11 (t, *J* = 6.5 Hz, 2H), 3.99 (t, *J* = 6.4 Hz, 2H), 3.92 (t, *J* = 6.4 Hz, 4H), 3.84 (s, 3H), 3.82 (t, *J* = 6.3 Hz, 8H), 1.91–1.78 (m, 4H), 1.77–1.66 (m, 12H), 1.63–1.24 (m, 112H), 0.89 (t, *J* = 6.8 Hz, 18H). ^13^C-NMR (100 MHz, CD_3_COCD_3_), δ (ppm): 167.0, 164.1, 156.5, 154.4, 144.8, 141.8, 135.1, 132.3, 127.5, 123.5, 116.1, 115.2, 103.7, 73.8, 69.7, 69.0, 68.8, 52.0, 32.8, 31.3, 30.6, 30.6, 30.6, 30.5, 30.5, 30.5, 30.5, 30.3, 30.3, 30.2, 30.2, 30.0, 29.9, 29.8, 29.6, 29.5, 29.3, 27.1, 27.0, 26.7, 26.6, 23.4, 14.4. IR (KBr, cm^−1^): 2919 (Csp^3^-H), 2850 (Csp^3^-H), 1716 (C=O), 1588 (arC-C), 1503 (arC-C), 1468 (arC-C), 1232 (C-O).

The obtained solid is dissolved in 1,4-dioxane (20 mL) and a solution of KOH (6.5 mmol, 0.36 g) in H_2_O (2 mL) is added. The reaction mixture is heated to 80 °C for 16 h. It is then cooled using an ice bath and acidified to pH = 3, using HCl 1M. The compound is extracted with ethyl acetate (3 × 50 mL). The organic layers are combined, washed with brine, dried over anhydrous MgSO_4_ and filtered. The solution is evaporated to dryness and the residue purified by recrystallization in ethanol.

A-TPA2: White solid. Yield: 69%. ^1^H-NMR (400 MHz, CD_2_Cl_2_), δ (ppm): 8.07–8.00 (m, 2H), 6.98–6.94 (m, 2H), 6.94–6.88 (m, 6H), 6.80–6.73 (m, 6H), 4.06 (t, *J* = 6.5 Hz, 2H), 3.93 (t, *J* = 6.4 Hz, 2H), 3.90 (t, *J* = 6.5 Hz, 4H), 1.91–1.69 (m, 8H), 1.60–1.51 (m, 4H), 1.49–1.22 (m, 36H), 0.88 (t, *J* = 6.9 Hz, 6H). ^13^C-NMR (100 MHz, CD_2_Cl_2_), δ (ppm): 171.6, 164.4, 155.2, 155.1, 142.5, 142.4, 132.8, 125.3, 125.2, 121.8, 115.6, 114.8, 68.9, 68.8, 68.7, 32.5, 30.2, 30.2, 30.2, 30.2, 30.0, 29.9, 29.9, 29.9, 29.6, 26.6, 26.4, 26.3, 23.3, 14.5. IR (KBr, cm^−1^): 3500–2400 (COOH), 2921 (Csp^3^-H), 2851 (Csp^3^-H), 1684 (C=O), 1605 (arC-C), 1502 (arC-C), 1472 (arC-C), 1257 (C-O), 1232 (C-O). MS (MALDI+, dithranol): 849.7 [M]^+^. Elemental analysis: calcd for (%) C_55_H_79_NO_6_: C 77.70, H 9.37, N 1.65; found: C 77.66, H 9.51, N 1.64.

A-TPA4: White solid. Yield: 78%. ^1^H-NMR (400 MHz, CD_2_Cl_2_), δ (ppm): 8.07–8.00 (m, 2H), 7.00–6.91 (m, 4H), 6.82–6.76 (m, 2H), 6.74 (d, *J* = 8.7 Hz, 2H), 6.60 (d, *J* = 2.6 Hz, 2H), 6.46 (dd, *J* = 8.6, 2.6 Hz, 2H), 4.06 (t, *J* = 6.5 Hz, 2H), 3.94 (t, *J* = 6.6 Hz, 2H), 3.91 (t, *J* = 6.7 Hz, 4H), 3.81 (t, *J* = 6.6 Hz, 4H), 1.90–1.64 (m, 12H), 1.60–1.51 (m, 4H), 1.50–1.21 (m, 72H), 0.92–0.85 (m, 12H). ^13^C-NMR (100 MHz, CD_2_Cl_2_), δ (ppm): 171.3, 164.3, 155.3, 150.4, 145.3, 143.1, 142.2, 132.8, 125.8, 121.9, 116.3, 115.7, 115.6, 114.8, 110.9, 70.5, 69.7, 68.9, 68.7, 32.5, 30.3, 30.3, 30.2, 30.1, 30.0, 30.0, 29.9, 29.6, 26.7, 26.6, 26.5, 26.4, 23.3, 14.5. IR (KBr, cm^−1^): 3500–2400 (COOH), 2920 (Csp^3^-H), 2851 (Csp^3^-H), 1690 (C=O), 1609 (arC-C), 1506 (arC-C), 1468 (arC-C), 1254 (C-O), 1232 (C-O). MS (MALDI+, dithranol): 1218.0 [M]^+^, 1241.0 [M+Na]^+^, 1263.0 [M-H+2Na]^+^. Elemental analysis: calcd for (%) C_79_H_127_NO_8_: C 77.85, H 10.50, N 1.15; found: C 77.51, H 10.30, N 1.31.

A-TPA6: White solid. Yield: 77%. ^1^H-NMR (400 MHz, CD_2_Cl_2_), δ (ppm): 8.07–8.00 (m, 2H), 7.05–6.99 (m, 2H), 6.98–6.93 (m, 2H), 6.83–6.77 (m, 2H), 6.20 (s, 4H), 4.06 (t, *J* = 6.5 Hz, 2H), 3.95 (t, *J* = 6.5 Hz, 2H), 3.87 (t, *J* = 6.6 Hz, 4H), 3.77 (t, *J* = 6.5 Hz, 8H), 1.91–1.76 (m, 4H), 1.75–1.64 (m, 12H), 1.61–1.53 (m, 4H), 1.50–1.21 (m, 108H), 0.92–0.85 (m, 18H). ^13^C-NMR (100 MHz, CD_2_Cl_2_), δ (ppm): 171.4, 164.3, 155.8, 153.8, 144.4, 141.4, 134.0, 132.8, 126.9, 121.9, 115.6, 114.8, 102.9, 74.0, 69.5, 68.8, 68.7, 32.5, 31.0, 30.4, 30.3, 30.3, 30.3, 30.0, 30.0, 29.7, 26.8, 26.7, 26.5, 26.4, 23.3, 14.5. IR (KBr, cm^−1^): 3500–2400 (COOH), 2921 (Csp^3^-H), 2851 (Csp^3^-H), 1690 (C=O), 1607 (arC-C), 1588 (arC-C), 1502 (arC-C), 1468 (arC-C), 1254 (C-O), 1237 (C-O). MS (MALDI+, dithranol): 1587.1 [M]^+^. Elemental analysis: calcd for (%) C_103_H_175_NO_10_: C 77.93, H 11.11, N 0.88; found: C 77.89, H 11.26, N 1.04.

## 4. Conclusions

Electro- and photoactive triarylamines with LC properties are achieved through the asymmetric substitution of the triphenylamine platform with dodecyloxy chains and one 4-(6-oxyhexyloxy)benzoic acid group. This last group allows the establishing of hydrogen bonding interactions in the crystal and LC phases. In the observed columnar LC phases, columns are formed, not by the stacking of a single molecule, but by hydrogen bonded aggregates. Compound A-TPA2, with two dodecyloxy chains, shows a hexagonal columnar mesophase stable at room temperature. In addition, it is able to show a photooxidation/assembly process under white light irradiation, similar to the one described for amide-containing triarylamines, but, in this case, without the assistance of hydrogen bonding amide functional groups.

## Figures and Tables

**Table 1:** Liquid crystal data of the triphenylamine-containing benzoic acids.

Compound	DSC Cycle	Phase Transition ^1^ T (°C) (ΔH (kJ mol^−1^))
A-TPA2	1st heating	Cr 62 (60.2) Iso
	1st cooling	Iso 53 (5.1) Col_h_
	2nd heating	Col_h_ 58 (5.0) Iso
	2nd cooling	Iso 53 (5.1) Col_h_
A-TPA4	1st heating	Cr 74 Cr’ 84 (86.8) ^2^ Iso
	1st cooling	Iso 45 (3.3) M 33 (41.8) Cr”
	2nd heating	Cr” 64 Cr 74 (58.3) ^2^ Iso
	2nd cooling	Iso 45 (3.3) M 33 (41.8) Cr”
A-TPA6	1st heating	Cr 53 Cr’ 58 (108.7) Iso
	1st cooling	Iso 33 M 25 (14.6) ^2^ Col_r_
	2nd heating	Col_r_ 34 M 37 (16.2) ^2^ Iso
	2nd cooling	Iso 33 M 25 (14.6) ^2^ Col_r_

^1^ Temperatures at the maximum of the peaks from DSC first heating and cooling and second heating cycle at a scanning rate of 10 °C min^−1^. Cr: crystal phase, Iso: isotropic liquid, Col_h_: hexagonal columnar mesophase, Col_r_: rectangular columnar mesophase, M: non-identified mesophase. ^2^ Enthalpy sum of two overlapped transitions.

**Table 2:** XRD data at 20 °C after cooling from the isotropic liquid.

Compound	Mesophase	Lattice Parameter (Å)	d_obs_ (Å)	d_calc_ (Å)	Miller Index
A-TPA2	Col_h_	*a* = 58.2	50.6	50.4	100
			29.5	29.1	110
			24.7	25.2	200
			19.2	19	210
			4.5 (diff)	-	-
A-TPA6	Col_r_	*a* = 111.2 *b* = 52.8	47.7	47.7	110
			26.4	26.4	20
			20.9	21.5	320
			16.9	16.8	230
			4.5 (diff)	-	-

**Table 3:** Electrochemical data and HOMO values.

Compound	E_1/2_^ox^ vs. Ag/AgCl (V)	E_1/2_^ox^ vs. Fc/Fc⁺ ^1^ (V)	HOMO ^2^ (eV)
A-TPA2	0.51	0.09	−4.89
A-TPA4	0.46	0.04	−4.84
A-TPA6	0.60	0.18	−4.98

^1^ E_1/2_^ox^ vs. Fc/Fc⁺ = E_1/2_^ox^ vs. Ag/AgCl − 0.43 V. ^2^ HOMO = −e (E_1/2_^ox^ vs. Fc/Fc⁺ + 4.8) eV.

## Data Availability

Data is contained within the article.
